# Integrin α3β1 Promotes Invasive and Metastatic Properties of Breast Cancer Cells through Induction of the Brn-2 Transcription Factor

**DOI:** 10.3390/cancers13030480

**Published:** 2021-01-27

**Authors:** Rakshitha Pandulal Miskin, Janine S. A. Warren, Abibatou Ndoye, Lei Wu, John M. Lamar, C. Michael DiPersio

**Affiliations:** 1 Department of Regenerative & Cancer Cell Biology, Albany Medical College, Albany, NY 12208, USA; Miskinr@amc.edu; 2 Department of Molecular & Cellular Physiology, Albany Medical College, Albany, NY 12208, USA; warrenj1@amc.edu (J.S.A.W.); lamarj@amc.edu (J.M.L.); 3 Department of Surgery, Albany Medical College, Albany, NY 12208, USA; Ndoyea@amc.edu (A.N.); Wul2@amc.edu (L.W.)

**Keywords:** triple-negative breast cancer, integrin α3β1, tumor cell invasion, metastasis, Brain-2, Brn-2, Oct-7, N-Oct3, POU3F2

## Abstract

**Simple Summary:**

Metastatic triple-negative breast cancer (TNBC) is highly lethal with limited therapy options. Integrin α3β1 is a cell surface receptor that interacts with the extracellular matrix and facilitates communication between tumor cells and their microenvironment. α3β1 is implicated in breast cancer progression and metastasis, so understanding mechanisms by which α3β1 promotes invasion and metastasis will facilitate the development of this integrin as a potential therapeutic target. Here we identify a novel role for α3β1 in promoting the expression of the transcription factor Brain-2 (Brn-2) in triple-negative breast cancer cells. We further report that Brn-2 promotes invasion and metastasis and partially restores invasion to cells in which expression of α3β1 has been suppressed. Bioinformatic analysis of patient datasets revealed a positive correlation between the expression of the genes encoding the integrin α3 subunit and Brn-2. In summary, our work identifies α3β1-mediated induction of Brn-2 as a mechanism that regulates invasive and metastatic properties of breast cancer cells.

**Abstract:**

In the current study, we demonstrate that integrin α3β1 promotes invasive and metastatic traits of triple-negative breast cancer (TNBC) cells through induction of the transcription factor, Brain-2 (Brn-2). We show that RNAi-mediated suppression of α3β1 in MDA-MB-231 cells caused reduced expression of Brn-2 mRNA and protein and reduced activity of the *BRN2* gene promoter. In addition, RNAi-targeting of Brn-2 in MDA-MB-231 cells decreased invasion in vitro and lung colonization in vivo, and exogenous Brn-2 expression partially restored invasion to cells in which α3β1 was suppressed. α3β1 promoted phosphorylation of Akt in MDA-MB-231 cells, and treatment of these cells with a pharmacological Akt inhibitor (MK-2206) reduced both Brn-2 expression and cell invasion, indicating that α3β1-Akt signaling contributes to Brn-2 induction. Analysis of RNAseq data from patients with invasive breast carcinoma revealed that high *BRN2* expression correlates with poor survival. Moreover, high *BRN2* expression positively correlates with high *ITGA3* expression in basal-like breast cancer, which is consistent with our experimental findings that α3β1 induces Brn-2 in TNBC cells. Together, our study demonstrates a pro-invasive/pro-metastatic role for Brn-2 in breast cancer cells and identifies a role for integrin α3β1 in regulating Brn-2 expression, thereby revealing a novel mechanism of integrin-dependent breast cancer cell invasion.

## 1. Introduction

Metastatic breast cancer is a highly lethal disease with only a small percentage of patients showing long-term remission in response to available therapies [1]. Triple-negative breast cancer (TNBC), a specific subtype that does not express estrogen receptor, progesterone receptor, or human epidermal growth factor receptor 2, has the worst overall survival outcomes, and it is the most likely subtype to metastasize to bone and lungs [2,3]. A better understanding of the underlying pathology of invasive disease and metastasis will be essential for the identification of novel therapeutic targets and the development of effective treatments for TNBC. The tumor microenvironment (TME) plays a critical role in regulating gene expression programs within tumor cells that drive malignant progression and invasion [4,5]. Tumor cell surface receptors that transmit cues from the TME to regulate gene expression are attractive therapeutic targets. Integrins, the major class of extracellular matrix (ECM) receptors, are heterodimeric transmembrane proteins that consist of an α and a β subunit and facilitate bi-directional signaling across the cell membrane to mediate communication between tumor cells and TME [6,7]. Integrins have emerged as key regulators of both cell-autonomous functions (e.g., proliferation, survival, migration) and cell-mediated modifications of ECM and other elements of the tissue microenvironment that are essential for both normal and pathological tissue remodeling, including tumor growth and progression [7,8,9]. However, further investigation is required to understand fully the mechanisms through which specific integrins regulate these processes and the gene expression programs that control them.

Integrin α3β1 is a laminin-binding integrin that is expressed in normal mammary epithelial cells and is often elevated in breast cancer [10]. The pleiotropic effects of this integrin in breast cancer implicate it as a potential therapeutic target [10,11]. Indeed, α3β1 promotes primary breast tumor formation and growth [12,13] and tumor cell invasion [13,14,15,16], and it plays a role in the induction of angiogenesis, stemness, and epithelial to mesenchymal transition [15]. Moreover, numerous studies have described cellular functions that are regulated by α3β1 and contribute to invasive tumor growth, as reviewed elsewhere [10], and studies using spontaneous or experimental metastasis models have shown that α3β1 promotes lung colonization by TNBC cells [17,18]. In addition to its established roles in regulating tumor cell-autonomous functions such as proliferation and migration [12,19,20], an emerging role for this integrin is the regulation of the tumor cell secretome that modifies the TME [19]. Much of this regulation appears to occur through α3β1-dependent regulation of gene expression that control some of these functions [13,20,21,22,23,24]. However, despite clear roles for α3β1 in the regulation of gene expression, its linkage to the regulation of specific transcription factors has been unknown.

In a previous study, we used an Affymetrix gene microarray platform to assess changes in the transcriptome of the human TNBC cell line, MDA-MB-231, in which α3β1 was suppressed through the stable expression of α3-targeting shRNA [23]. Our analysis identified the mRNA that encodes Brain-2 (Brn-2/Oct-7/N-Oct3/POU3F2) among the top 15 transcripts that were reduced in α3 knockdown (α3-KD) cells. Brn-2 is a neural transcription factor with important roles in the development of the central nervous system [25]. In the context of cancer, Brn-2 is overexpressed in glioblastoma [26], promotes cell growth and neuroendocrine differentiation in small cell lung cancer [27] and prostate cancer [28], and is a major driver of invasion and metastasis in melanoma [29]. Although a recent study implicated Brn-2 as a potential regulator of breast cancer genes [30], a causal role for Brn-2 in breast cancer invasion or metastasis remains unknown.

In the current study, we hypothesized that α3β1-dependent regulation of Brn-2 contributes to the invasive and metastatic properties of breast cancer cells. Using established models of invasion and experimental metastasis, we confirmed that α3-KD TNBC cells display reduced invasive properties in vitro and reduced lung colonization in vivo, respectively, consistent with pro-invasive/pro-metastatic roles for α3β1. Quantitative PCR (qPCR) and Western blot analysis demonstrated that RNAi-mediated suppression of α3β1 in two TNBC lines, MDA-MB-231 and SUM159, leads to decreased Brn-2 expression, validating our preliminary finding in gene expression arrays [23]. Analysis of publicly available RNAseq datasets from breast cancer patients revealed that high *BRN2* gene expression is correlated with poor survival. Moreover, *BRN2* expression is significantly upregulated in patients with basal-like (i.e., triple-negative) breast cancer, where it also correlates with high expression of *ITGA3* mRNA. siRNA-mediated suppression of Brn-2 in MDA-MB-231 cells revealed a pro-invasive role, and exogenous Brn-2 expression partially rescued the invasion deficiency seen in α3-KD cells. Using a pharmacological approach, we identified α3β1 signaling through Akt as a contributing pathway to Brn-2 induction. Collectively, our findings show that integrin α3β1 induces Brn-2 to promote invasive and metastatic properties of TNBC cells, and they implicate this regulation in the progression of human TNBC.

## 2. Results

### 2.1. Suppression of Integrin α3β1 in MDA-MB-231 Cells Decreases the Potential for Lung Colonization

**Figure 1:**
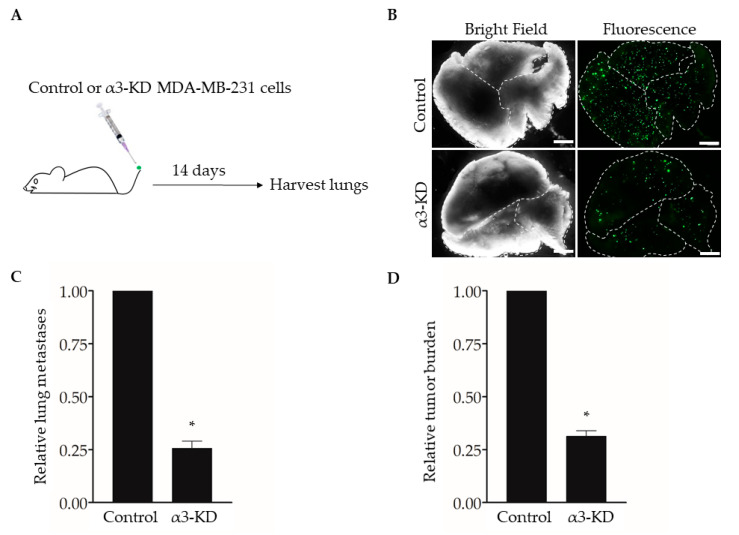
RNAi-targeting of the integrin α3 subunit decreases lung colonization by MDA-MB-231 cells. (**A**) MDA-MB-231 cells that stably express either non-targeting shRNA (Control) or α3-targeting shRNA (α3-KD) were labeled fluorescently by transduction with a lentivirus expressing ZsGreen (pHAGE- IRES- ZsGreen). A total of 5 × 10^4^ cells were injected into the tail-vein of five-week-old, female NSG™ mice, and lungs were harvested 14 days later. (**B**) Representative bright field and fluorescent images of lungs from mice injected with either control or α3-KD cells, as indicated. Dashed lines outline individual lobes. (**C**,**D**) Graphs showing (**C**) the relative number of lung metastases or (**D**) relative tumor burden, normalized to the average of the control. *n* = 6 (Control) or 7 (α3-KD); mean +/− SEM; * *p* < 0.05; two-tailed *T*-test; scale bar, 2 mm. Similar results were obtained in a separate experiment using seven-month-old, female NSG™ mice (*n* = 4 mice/group).

Studies using human or mouse TNBC cell lines have shown that expression of integrin α3β1 promotes cell invasion in vitro [13,14,15] and spontaneous metastasis and lung colonization in vivo [17]. To confirm this effect of suppressing α3β1 in our model, we used an experimental metastasis approach of tail vein injection [31]. We previously derived MDA-MB-231 cells that stably express either a non-targeting shRNA (control) or an shRNA that knocks down the mRNA transcript for the α3 integrin subunit (α3-KD) [13]. As the α3 subunit pairs exclusively with the β1 subunit [6], α3 knockdown leads to the effective suppression of integrin α3β1 [13]. Control or α3-KD cells were fluorescently labeled by transduction with a lentivirus that expresses ZsGreen, then injected into the tail veins of NSG™ mice, and lungs were harvested after 14 days to assess metastatic burden (Figure 1A,B). Compared with lungs harvested from control mice, lungs from mice injected with α3-KD cells showed a significant reduction in both the number of colonies (Figure 1C) and total tumor burden (Figure 1D), showing that suppression of α3β1 leads to a decrease in lung colonization.

### 2.2. Integrin α3β1 Regulates Expression of the Brn-2 Transcription Factor in Triple-Negative Breast Cancer Cells

**Figure 2:**
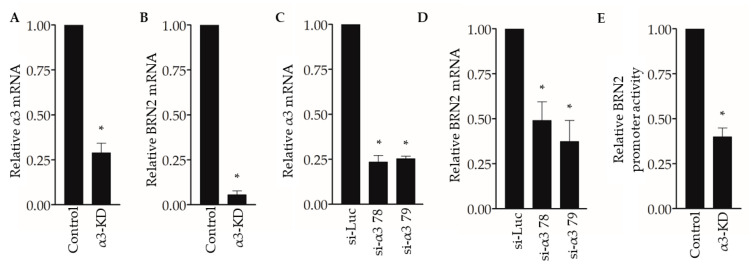
Suppression of integrin α3β1 decreases *BRN2* mRNA and promoter activity in MDA-MB-231 cells. (**A**,**B**) qPCR showing (**A**) relative integrin α3 and (**B**) *BRN2* mRNA levels in MDA-MB-231 cells stably transduced with α3-targeting shRNA (α3-KD), relative to control cells that express non-targeting shRNA (Control). (**C**,**D**) qPCR showing (**C**) integrin α3 and (**D**) *BRN2* mRNA levels in MDA-MB-231 cells transfected with siRNA that targets integrin α3 (si-α3 78 or si-α3 79) relative to cells transfected with control siRNA (si-Luc). (**E**) *BRN2* promoter/luciferase reporter activity in α3-KD cells relative to control cells. *n* = 3; mean +/− SEM; * *p* < 0.05; two-tailed *T*-test (**A**,**B**,**E**) or two-tailed *T*-test with Bonferroni correction (**C**,**D**).

To begin exploring the underlying mechanisms through which suppression of α3β1 reduces pro-invasive/pro-metastatic properties of breast cancer cells, we revisited our previously published gene microarray study to compare the transcriptomes of control and α3-KD MDA-MB-231 cells [23]. Our analysis identified the mRNA that encodes the transcription factor Brain-2 (Brn-2/Oct-7/N-Oct3/POU3F2) among the top 15 transcripts that were reduced in α3-KD cells compared with control cells, which we confirmed by qPCR analysis (Figure 2A,B). We further demonstrated α3β1-dependent expression of *BRN2* mRNA by transiently suppressing α3β1 using two distinct siRNAs that target the α3 integrin subunit (Figure 2C,D). Interestingly, transfection with a *BRN2* promoter-driven luciferase reporter plasmid revealed that the *BRN2* promoter was significantly less active in α3-KD cells compared to control cells (Figure 2E), suggesting that reduced promoter activity contributes to reduced *BRN2* mRNA.

**Figure 3:**
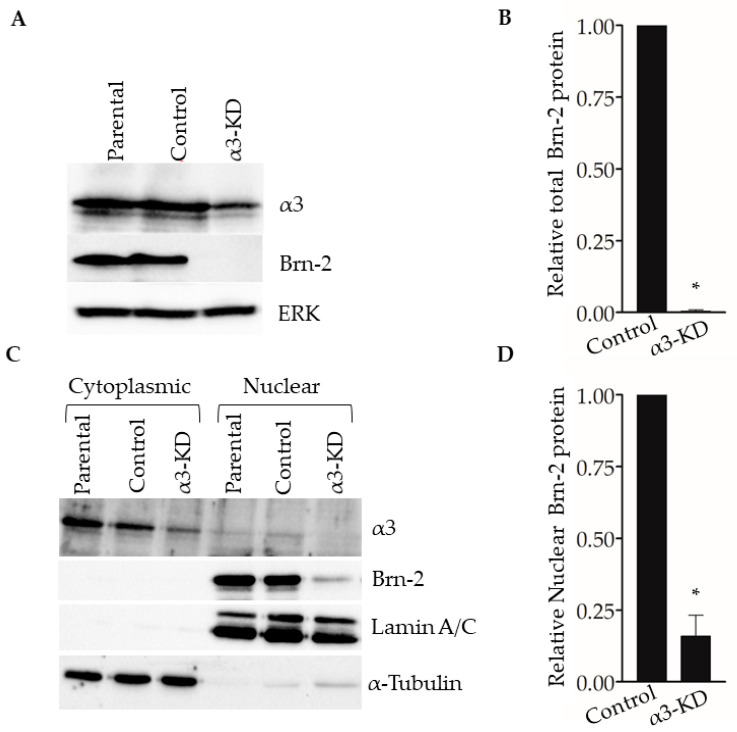
Suppression of integrin α3β1 in MDA-MB-231 cells decreases Brn-2 protein levels. (**A**) Representative Western blot showing integrin α3, Brn-2, and ERK (loading control) protein levels in whole-cell lysates obtained from MDA-MB-231 parental (i.e., non-transduced) cells, or cells expressing either non-targeting (Control) or α3-targeting (α3-KD) shRNA. (**B**) Relative Brn-2 protein in α3-KD cells normalized to control. (**C**) Representative Western blot showing subcellular localization of Brn-2 protein. Cytoplasmic and nuclear fractions were obtained from parental, control, and α3-KD cells. α-Tubulin and LaminA/C served as fractionation markers. (**D**) Relative nuclear Brn-2 protein in α3-KD cells normalized to control. *n* = 3; mean +/− SEM; * *p* < 0.05; two-tailed *t*-test.

Western blotting of whole-cell lysates showed that Brn-2 protein was decreased dramatically in α3-KD cells relative to control (Figure 3A,B). Assessment of subcellular fractions revealed that Brn-2 protein was detected primarily in the nuclear fraction of parental (i.e., non-transduced) or control MDA-MB-231 cells, as expected [32], and that it was reduced substantially within this fraction of α3-KD cells (Figure 3C,D). We observed similarly reduced Brn-2 protein following stable shRNA-mediated α3 suppression in a distinct lung metastatic variant of the MDA-MB-231 cell line, TGL-4175 [33] (Figure S1A,B), and following transient siRNA-mediated α3 suppression in a second TNBC cell line, SUM159 (Figure S1C–E), showing that α3β1-dependent *BRN2* gene expression occurs in distinct TNBC cell lines.

### 2.3. ITGA3 mRNA and BRN2 mRNA Are Positively Correlated in Triple-Negative Breast Cancer Patients

**Figure 4:**
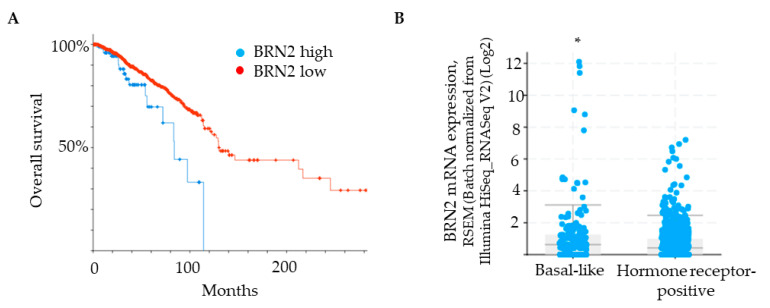
*BRN2* expression positively correlates with poor patient survival and is higher in patients with a basal-like subtype of breast cancer. (**A**) Kaplan–Meier plot showing that overall survival of breast cancer patients is correlated with *BRN2* expression (0.1 FPKM cut-off; *p*-value, 3.651 × 10^−3^. (**B**) *BRN2* gene expression in breast cancer tumors grouped as basal-like or hormone receptor-positive (*p*-value, 1.644 × 10^−3^). * *p* < 0.05.

Brn-2 has been reported to be a major driver of invasion and metastasis in melanoma [29]. Although Brn-2 has also been associated with breast cancer [30], a functional role in breast cancer cells remains unclear. We used a bioinformatic approach [cBioPortal; [34,35]] to determine if *BRN2*/*POU3F2* gene expression correlates with breast cancer patient survival and compare *BRN2* gene expression between hormone receptor-positive and basal-like subtypes of breast cancer. Patient data were obtained from the Human Protein Atlas, which uses the Breast Invasive Carcinoma dataset (TCGA, PanCancer Atlas). A Kaplan–Meier plot shows that survival of patients with low *BRN2*/*POU3F2* expression in tumors is significantly greater than that of patients with high *BRN2* expression (Figure 4A). Grouping of samples into hormone receptor-positive versus basal-like subtypes, which are generally triple-negative, revealed that the latter group expresses significantly higher levels of *BRN2* mRNA (Figure 4B). Moreover, the analysis of patients with basal-like tumors revealed a significant, positive correlation between the expression of the *ITGA3* gene, which encodes the α3 integrin subunit, and the *BRN2* gene (*p*-adj = 1.24 × 10^−5^; see Materials and Methods for details of the analysis). Since the α3 integrin subunit partners exclusively with the β1 integrin subunit [6], this correlation indicates a positive association of integrin α3β1 with Brn-2 expression in clinical samples of basal-like tumors.

### 2.4. RNAi-Mediated Suppression of BRN2 in Breast Cancer Cells Reduces Invasion In Vitro and Lung Colonization In Vivo

**Figure 5:**
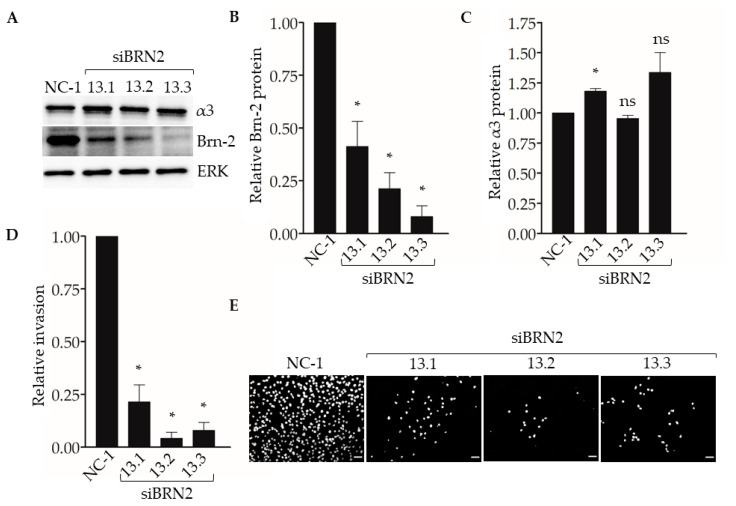
RNAi-targeting of *BRN2* reduces MDA-MB-231 cell invasion. Matrigel transwell invasion assays were performed using MDA-MB-231 cells transfected with either non-targeting dicer-substrate siRNA (NC-1) or *BRN2*-targeting dicer-substrate siRNA (si*BRN2* 13.1, 13.2, or 13.3). Nuclei were stained with DAPI, imaged, and quantified. (**A**) Representative Western blots for Brn-2, integrin α3, and ERK (loading control); results for Brn-2 and α3 are quantified in (**B**) and (**C**), respectively. (**D**) Graph shows invasion of Brn-2 knockdown cells relative to control cells; *n* = 3; mean +/− SEM; * *p* < 0.05; two-tailed *T*-test with Bonferroni correction. (**E**) Representative images of DAPI-stained cells that invaded the undersides of transwell filters. Scale bar, 100 µm.

To determine if suppression of Brn-2 leads to reduced invasion of breast cancer cells, we performed Matrigel transwell invasion assays using MDA-MB-231 cells that express *BRN2*-targeting dicer-substrate siRNA. Compared with control non-targeting dicer-substrate siRNA, transfection with each of three distinct *BRN2*-targeting dicer-substrate siRNAs caused efficient suppression of Brn-2 protein without substantially affecting α3 protein levels (Figure 5A–C). Moreover, transfection with each *BRN2*-targeting siRNA caused significantly decreased cell invasion compared with control siRNA (Figure 5D,E). We observed similar results in the SUM159 cells (Figure S2A,B).

**Figure 6:**
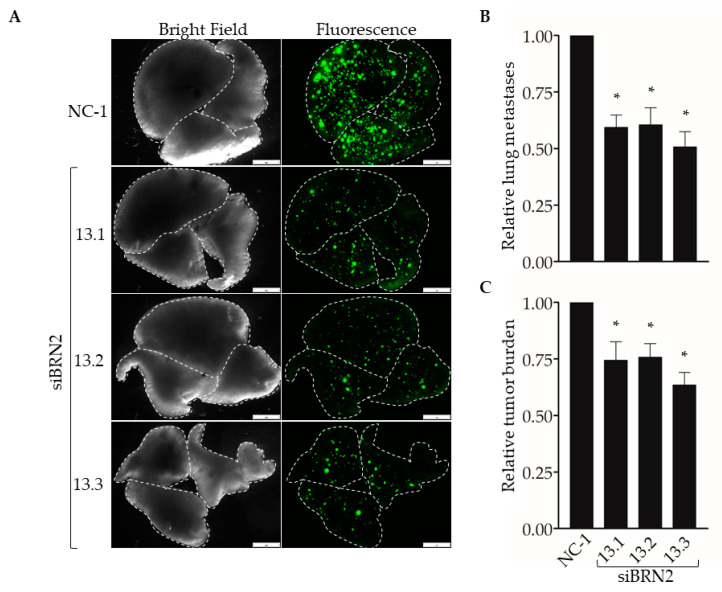
RNAi-targeting of *BRN2* decreases lung colonization by MDA-MB-231 cells. A total of 5 x 10^4^ cells stably transduced with ZsGreen and transiently expressing either non-targeting dicer-substrate siRNA (NC-1) or *BRN2*-targeting dicer-substrate siRNA (si*BRN2* 13.1, 13.2, or 13.3) were injected into tail-veins of NSG™ mice. (**A**) Representative bright-field or fluorescent (ZsGreen) images of lungs harvested from mice 14 days post-injection. Dashed lines outline individual lobes. (**B**,**C**) Graphs show (**B**) relative lung metastases and (**C**) tumor burden in lungs of mice injected with control or Brn-2 knockdown cells; *x*-axis labels in (**C**) apply to (B); *n* = 7 per group; mean +/− SEM; * *p* < 0.05; two-tailed *t*-test with Bonferroni correction; scale bar, 2 mm.

Next, we used our experimental metastasis model to determine the effect of suppressing Brn-2 expression on lung colonization (Figure 6A). When compared with MDA-MB-231 cells transfected with the control dicer-substrate siRNA, cells transfected with each of the three *BRN2*-targeting dicer-substrate siRNAs showed a significant decrease in the number of lung colonies (Figure 6B) and total metastatic burden (Figure 6C). Together, results in Figure 5 and Figure 6 support a pro-invasive and pro-metastatic role for Brn-2. 

### 2.5. Restoring Brn-2 Expression in α3β1-Deficient MDA-MB-231 Cells Partially Rescues the Invasive Phenotype

**Figure 7:**
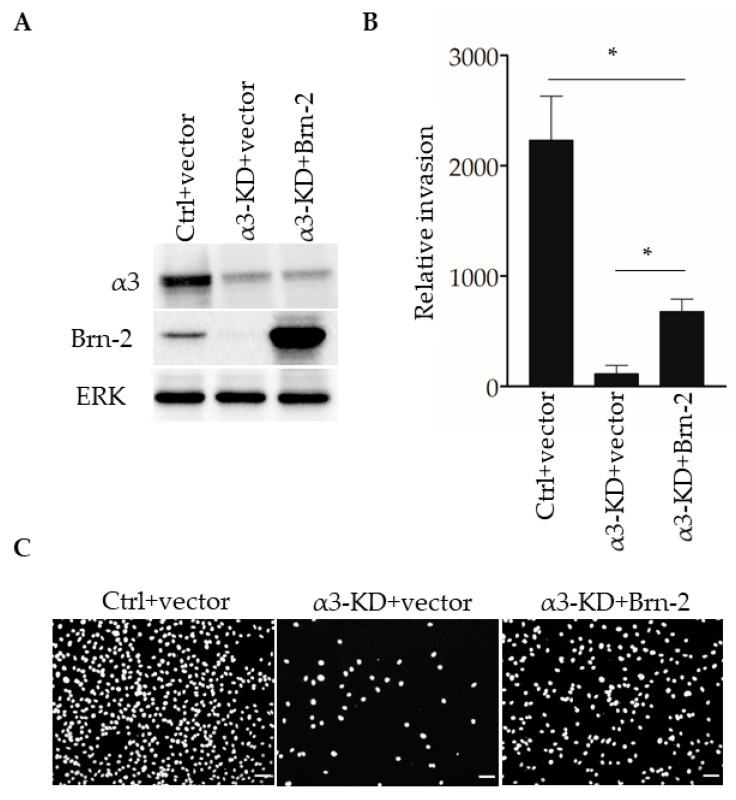
Brn-2 over-expression partially rescues decreased invasion of α3-knockdown MDA-MB-231 cells. (**A**) Representative Western blots of control (Ctrl) or α3-KD MDA-MB-231 cells transiently expressing either pHAGE-ZsGreen (+vector) or pHAGE-*BRN2*-ZsGreen (+Brn-2) expression plasmid. (**B**) Graph shows relative invasion of α3-KD cells overexpressing Brn-2 compared to vector alone; *n* = 3; mean +/− SEM; * *p* < 0.05; two-tailed *T*-test with Bonferroni correction. (**C**) Representative images of invaded cells. Scale bar, 100 µm.

We previously reported that stable shRNA-mediated suppression of α3β1 in MDA-MB-231 cells leads to reduced invasion in vitro [13]. To determine whether the expression of exogenous Brn-2 restores invasion to these cells, we transfected α3-KD MDA-MB-231 cells with an expression plasmid for mouse Brn-2, or with the parental vector as a control, then performed transwell invasion assays. Brn-2-transfected α3-KD cells, which expressed exogenous Brn-2 at a considerably higher level than that of endogenous Brn-2 in the parental cell line (Figure 7A), showed greater invasion compared with control-transfected α3-KD cells (Figure 7B,C). Interestingly, however, invasion in these cells was restored to only about 50% of the level seen in parental (i.e., α3β1-expressing) MDA-MB-231 cells (Figure 7B), suggesting that α3β1 also promotes invasion through mechanisms that are independent of Brn-2. Together with our above finding that Brn-2 is necessary for the full invasive potential of α3β1-expressing MDA-MB-231 cells (Figure 5), this result suggests that α3β1-dependent induction of Brn-2 promotes the invasive phenotype.

### 2.6. Integrin α3β1-Dependent Phosphorylation of Akt Contributes to the Induction of Brn-2 Expression and Cell Invasion

**Figure 8:**
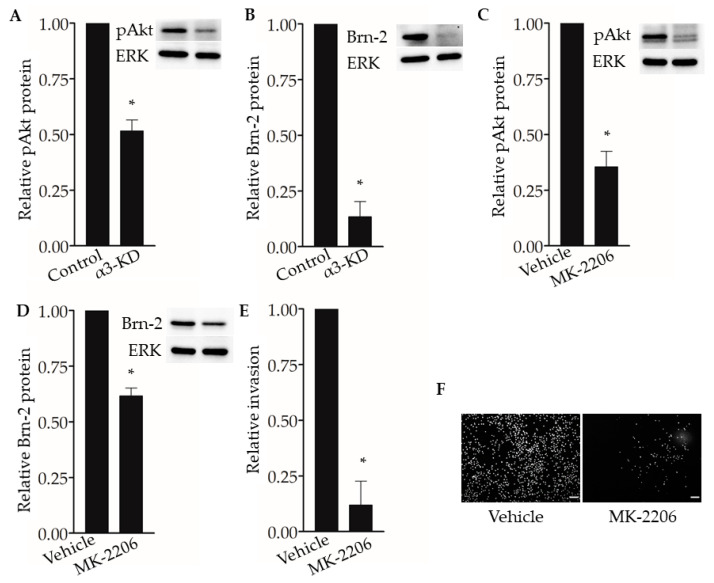
Integrin α3β1-dependent Akt phosphorylation influences Brn-2 expression and invasion of MDA-MB-231 cells. (**A**,**B**) Representative Western blots and corresponding graphs show relative levels of (**A**) phospho-Akt (pAkt) and (**B**) Brn-2 in α3-KD cells relative to control. (**C**,**D**) Representative Western blots and corresponding graphs show relative levels of (**C**) pAkt and (**D**) Brn-2 in cells treated with DMSO (vehicle) or pAkt inhibitor (MK-2206). (**E**) Graph shows the relative invasion of MK-2206-treated cells compared to vehicle-treated cells. (**F**) Representative images for data in (**E**). *n* = 3 experiments; mean +/− SEM; * *p* < 0.05; two-tailed *T*-test; scale bar, 100 µm.

**Figure 9:**
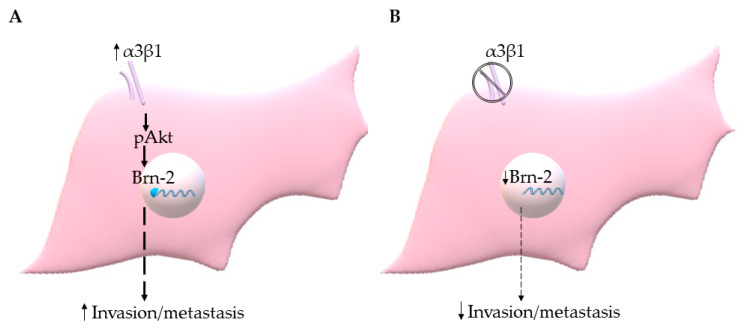
(**A**) Integrin α3β1 signaling through Akt induces Brn-2 to stimulate invasion/metastasis of TNBC cells. (**B**) Suppression of α3β1 leads to reduced Brn-2, which in turn results in the decreased invasive and metastatic potential of TNBC cells.

The PI3K-Akt signaling pathway has been shown to regulate Brn-2 expression in melanoma cells [29,36], and integrin α3β1 has been shown to activate this pathway in epithelial cells and cancer cells, including breast cancer cells [15,16,37,38]. These previous findings prompted us to investigate whether α3β1-dependent Akt activation contributes to the induction of Brn-2 in MDA-MB-231 cells. Western blotting showed that, compared with α3β1-expressing control cells, α3-KD cells showed a reduced level of phospho-Akt (pAkt) (Figure 8A) that was correlated with reduced Brn-2 levels (Figure 8B). To determine whether Akt activation is causally linked to Brn-2 expression, we utilized a highly selective Akt inhibitor, MK-2206. Treatment of parental (i.e., α3β1-expressing) MDA-MB-231 cells with MK-2206 significantly decreased the levels of both pAKT (Figure 8C) and Brn-2 protein (Figure 8D) and also caused a substantial reduction of cell invasion (Figure 8E,F). Although total Akt levels tended to be lower in α3-KD cells relative to control, this decrease did not achieve statistical significance (Figure S3A), and total Akt levels did not change upon MK-2206 treatment (Figure S3B). Taken together, our findings suggest that integrin α3β1 enhances breast cancer cell invasion through activation of an Akt signaling pathway and that this effect occurs partly through induction of Brn-2 (Figure 9A,B).

## 3. Discussion

Integrins have critical roles throughout cancer progression, including local invasion of the primary tumor and at several steps of the metastatic cascade (reviewed in [9,39]). Previous studies from our group and others have established that integrin α3β1 promotes invasive properties of breast cancer cells [10,13,14,15]. Moreover, our current results using an experimental metastasis model showed that RNAi-mediated suppression of α3β1 decreases lung colonization by MDA-MB-231 cells, consistent with a previous report that an α3β1-blocking antibody decreased pulmonary arrest of this TNBC line [18] and supporting a pro-metastatic role for α3β1. While these findings suggest that blocking α3β1 is an attractive strategy [10], the development of this integrin as a therapeutic target requires a better understanding of the mechanisms through which it promotes tumor cell invasion and metastasis.

Among the α3β1-regulated cellular processes that can affect tumor growth and progression is its ability to promote pro-tumorigenic/pro-invasive gene expression programs that control the tumor cell secretome, ECM remodeling, and other cell functions [10,19,22]. As transcription factors play critical roles in the regulation of gene expression, it is not surprising that alterations in the expression or activity of key transcription factors have been linked to the development of several cancers [40]. While it has long been known that signaling downstream of integrins can influence gene expression through the regulation of transcription [41,42,43,44,45,46,47,48,49], roles for distinct integrins in the regulation of specific transcription factors remain underexplored.

In the current study, we identified a novel role for integrin α3β1 in the regulation of the transcription factor Brn-2, and we linked this regulation to the invasive and metastatic properties of TNBC cells. Brn-2 is a major gene regulator during the development of the central nervous system [25,50]. It is also expressed in several cancers ([26,27,28]), and in melanoma, it is a major driver of the invasive phenotype [29]. However, a functional role for Brn-2 in breast cancer has not been reported previously. Here, we showed that suppression of α3β1 in TNBC cells, which is achieved through RNAi-mediated targeting of the α3 integrin subunit, caused a decrease in Brn-2 expression. A pro-invasive/pro-metastatic role for Brn-2 in breast cancer cells is supported by our findings that siRNA-targeting of Brn-2 in MDA-MB-231 cells led to decreased invasion in vitro and reduced lung colonization in vivo. Reduced invasive potential indicates that Brn-2 may promote the extravasation step in the experimental metastasis model, although we cannot rule out a role for Brn-2 in promoting cell proliferation and/or survival following the seeding of lung colonies. Interestingly, exogenous expression of Brn-2 in α3-KD MDA-MB-231 cells only partially rescued invasion, suggesting that α3β1-dependent regulation of Brn-2 is one of several mechanisms through which this integrin promotes cell invasion.

Our analysis of breast cancer patient data from the Breast Invasive Carcinoma dataset (TCGA, PanCancer Atlas) revealed a significant, positive correlation between high expression of the *BRN2/POU3F2* gene and poor patient survival. Interestingly, *BRN2* expression was significantly higher in the basal-like subtype of breast cancer, which is generally triple-negative. Our additional observation that high *ITGA3* expression was positively correlated with high *BRN2* expression in basal-like breast cancer lends credence to the clinical relevance of our experimental findings that α3β1 induces Brn-2 expression in TNBC cells.

Previous studies have identified several intracellular signaling pathways through which Brn-2 can be induced, including pathways that signal through β-catenin, MAPK, or PI3K-Akt [29,36,51]. In particular, the PI3K-Akt pathway is a critical regulator of Brn-2 expression in melanoma cells [29,36], and integrin-dependent activation of this pathway has long been known to promote breast cancer cell invasion [16,52]. Indeed, several studies have shown that integrin α3β1 stimulates the PI3K-Akt pathway, including in MDA-MB-231 cells [15,16]. Our current findings that α3-KD MDA-MB-231 cells showed reduced phosphorylation of Akt compared with control cells, and that treatment of the α3β1-expressing parental cells with an Akt-specific pharmacological inhibitor decreased both Brn-2 protein levels and invasion, suggest that α3β1 signals, at least partly, through PI3K-Akt to induce Brn-2.

To our knowledge, ours is the first study to demonstrate integrin-mediated regulation of Brn-2 or to identify a specific transcription factor as a key pathway component in α3β1-dependent effects on cell invasion. Future investigations will identify downstream target genes through which Brn-2 promotes invasion of breast cancer cells. Possible mechanisms include Brn-2-dependent regulation of Notch signaling effectors, as reported in melanoma cells [53]. In addition to its ability to regulate gene expression, α3β1 regulates several other tumor cell functions that are known to contribute to invasion and metastasis, including cell adhesion to laminins, interactions with the CD151 tetraspanin protein, and ECM proteolysis (reviewed in [11,54,55]). The extent to which some of these other functions are linked to α3β1-dependent changes in gene expression remains to be seen. In any case, the ability of α3β1 to induce Brn-2, together with the pleiotropic functions of this integrin on tumor cells, enhances its potential value as a therapeutic target for TNBC.

## 4. Materials and Methods

### 4.1. Cell Culture

MDA-MB-231 cells (American Type Culture Collection, ATCC, Manassas, VA, USA) were cultured in Dulbecco’s Modified medium (DMEM) (Ref 10-013-CV, Corning, Waltham, MA, USA) and supplemented with 10% fetal bovine serum (Cat. 100–106, Gemini Bio-Products, West Sacramento, CA, USA) and 1% L-glutamine (Ref. 25030-081, Gibco, Waltham, MA, USA). Control and α3-KD variants of this line were established previously in our lab [13] and authenticated by STR-profiling (ATCC Cell Line Authentication Service). SUM159 cells (Asterand, Detroit, MI, USA) were cultured as previously described [56]. All cell lines were negative for the presence of mycoplasma contamination using an established qPCR test [57]. For signaling studies, cells were treated with the pAkt inhibitor, MK-2206 (Cat. # S1078, Selleck Chemicals, Houston, TX, USA), at a final concentration of 3 µM for 48 h.

### 4.2. Experimental Metastasis Assay

MDA-MB-231 cells were fluorescently labeled by stable transduction with a lentivirus expressing ZsGreen (pHAGE-IRES-ZsGreen). A total of 5 × 10^4^ cells were injected into the tail-vein of female, five-weeks-old NSG™ mice (Jackson Laboratories, stock# 005557-NOD.Cg-Prkdc<scid>II2rg<tm1Wjl>SzJ, Bar Harbor, ME, USA) as described in [31]. Lungs were harvested 14-days post-injection and imaged using Leica M205 FA & Lecia DCF3000 G (GFP and bright field filters) from Leica Microsystems (Wetzlar, Germany). Fiji ImageJ software was used to quantify the number of lung metastases and tumor burden. Briefly, images were individually thresholded to accurately represent the green fluorescence of tumor colonies and counted using the analyze particles option. For metastatic burden measurements, bright field images of the lung lobes were used to create regions of interest and applied to the thresholded fluorescent images to measure the percentage area of fluorescence. For figures, brightness and contrast were optimized identically for all panels. All animal work was approved by the Institutional Animal Care and Use Committee at Albany Medical College (project number: 20-05002).

### 4.3. Cloning

All plasmids (pHAGE-IRES-ZsGreen and pHAGE-Brn-2-IRES-ZsGreen) were generated using standard molecular biology techniques. *BRN2* cDNA was PCR amplified from commercially available plasmid CAG-m*BRN2* (a gift from Connie Cepko, Harvard University, Cambridge, MA, USA) (Addgene plasmid # 19711; http://n2t.net/addgene:19711; RRID:Addgene_19711), [58]).

### 4.4. siRNA Transfection

siRNAs were introduced into cells using Lipofectamine™ 2000 (Ref. 11668-019, Invitrogen, Waltham, MA) for 21-mer siRNA or RNAiMax (Ref. 13778-075, Invitrogen) for dicer-substrate siRNA following manufacturer’s protocols. Briefly, a final concentration of 10 nM siRNA (anti-luciferase (Cat. D-002050-01-20, Dharmacon™, Lafayette, CO, USA), Mission siRNA universal negative control #2 (SIC002, Sigma-Aldrich, St. Louis, MO, USA) or anti-integrin α3 subunit (Cat. SASI_Hs01_00196578, SASI_Hs01_00196579, Millipore Sigma, St. Louis, MO), or dicer-substrate non-targeting control (cat. 51-01-14-03, IDT, Coralville, IA, USA) or *BRN2*-targeting siRNA (hs.Ri.POU3F2.13.1, hs.Ri.POU3F2.13.2, hs.Ri.POU3F2.13.3, IDT)) were incubated with Lipofectamine diluted in OptiMEM (31985070, Gibco, Waltham, MA, USA) and transferred into tissue culture treated 12-well plates. A total of 1 × 10^5^ cells were plated and harvested after 72 h of siRNA transfection.

### 4.5. DNA Transfection and Dual-Luciferase Reporter Assay

Cells were reverse-transfected with the *BRN2* promoter reporter (pGL410-*BRN2*p, a gift from Robert Judson-Torres, University of Utah, Salt Lake City, UT, USA) (Addgene plasmid # 110733; http://n2t.net/addgene:110733; RRID:Addgene_110733) [59] using Lipofectamine LTX transfection reagent (Ref. 15338-100, Invitrogen) following the manufacturer’s protocol, then harvested 72 h later for assessment of luciferase reporter expression. To control for differences in transfection efficiency, signals were normalized to a co-transfected cytomegalovirus (CMV) promoter-driven Renilla plasmid [60]. Dual-luciferase reporter assay was performed as per the manufacturer’s protocol (Ref. E1910, Promega, Madison, WI, USA) and luciferase signal read on the SpectraMax i3 (I3-P/i3 Multi-Mode Microplate/EA, Molecular Devices, San Jose, CA, USA).

### 4.6. RNA Isolation and qPCR

RNA was isolated using the Trizol Reagent (Cat. number 15596-018, Life Technologies, Waltham, MA, USA) following the manufacturer’s protocol, then DNAse treated using Turbo DNA-free™ Kit (Cat. AM1907, Ambion, Waltham, MA, USA). RNA quality was assessed using a Nanodrop (NanoDrop 1000 Spectrophotometer, Thermofisher, Waltham, MA, USA). cDNA was synthesized using iScript™ cDNA Synthesis Kit (Cat. 1708890, Bio-Rad, Hercules, CA, USA), and qPCR was performed using SsoAdvanced™ Universal SYBR^®^ Green Supermix (Cat. 172-5270, Bio-Rad) in the Bio-Rad CFX96 Touch thermocycler using the following conditions: 95 °C 3 min, 1 cycle; followed by (95 °C 10 s, 59 °C 30 s), 39 cycles; specificity of qPCR reactions were assessed using melt curve analysis (60 °C to 95 °C, 0.5 °C increments). Reference genes were selected by testing the stability of expression (M-score) of common house-keeping transcripts in a commercially available pre-designed reference gene plate (Reference genes H96, Bio-Rad) using cDNA from control and α3-KD cells. M-score analysis identified PSMC4, PUM1, and IPO8 as transcripts with the highest stability between our control and experimental (α3-KD) conditions. The geometric mean of these three reference genes was used for normalization. qPCR primers were designed using IDT PrimerQuest^®^ tool (IDT, Coralville, IA, USA); primer sequences are as follows-integrin α3 Fwd-GCAGGTAATCCATGGAGAGAAG, Rev-CCACTAGAAGGTCTGGGTAGAA; PSMC4 Fwd-GGAGGTTGACTTGGAAGACTATG, Rev-GACAGCCAACATTCCACTCT; PUM1 Fwd-TACGTGGTCCAGAAGATGATTG, Rev-GCCATAGGTGTACTTACGAAGAG; IPO8 Fwd-CATGATGCCTCTCCTGCATAA, Rev-CTTCTCCTGCATCTCCACATAG; *BRN2* Fwd-ATGGGAACTGGCCTTTAGTG, Rev-GCTTCTGACCTTACCTACTTGG. Melt curves and Ct values were accessed using Bio-Rad CFX Manager software (Bio-Rad, Hercules, CA, USA).

### 4.7. Western Blotting

Whole-cell lysates were harvested in lysis buffer (9803, Cell Signaling, Waltham, MA, USA) supplemented with protease inhibitor (Ref. 11836170001, Roche, St. Louis, MO, USA), 0.1% Sodium dodecyl sulfate, and 0.5% sodium deoxycholate; samples were sonicated for 15 s. Cytoplasmic and nuclear fractions were obtained following the manufacturer’s protocol for NE-PER Nuclear and Cytoplasmic Extraction Reagents (Cat. 78833, Thermo Scientific, Waltham, MA, USA). Briefly, cell pellets were lysed in CER I and CER II on ice and centrifuged to obtain cytoplasmic (supernatant) and nuclear (pellet) fractions. The nuclear pellet was lysed in NER on ice to obtain the nuclear fraction. For adhesion-dependent signaling experiments, 1 × 10^5^ cells in serum-free DMEM media were incubated on ice for 30 min before plating onto tissue culture treated six-well plates. Cells were incubated at 37 °C for 3 h and lysed in lysis buffer supplemented with phosphatase inhibitor (1:100) (Cat. 524625, Calbiochem, Burlington, MA, USA).

Protein quantification was performed using Pierce™ BCA Protein Assay Kit (Cat. 23227, Thermo Scientific, Rockford, IL, USA). Samples were run on homemade 10% SDS-PAGE gels, transferred to nitrocellulose membranes, blocked in 5% bovine serum albumin, and incubated overnight at 4 °C with the primary rabbit antibodies against Brn-2 (#12137, Cell Signaling, Waltham, MA, USA), integrin α3 [61], ERK2 (sc-154, Santa Cruz, Dallas, TX, USA), phospho-Akt (ser473) (9271, Cell Signaling), Akt (9272, Cell Signaling), or Lamin A/C (2032, Cell Signaling), or with primary mouse antibody against α-tubulin (T9026, Sigma-Aldrich, St. Louis, MO, USA), followed by incubation at room temperature for 1 h with HRP-crosslinked goat anti-rabbit (7074, Cell signaling) or goat anti-mouse (Cat. 62-6520, ThermoFisher, Waltham, MA, USA) as appropriate. Blots were treated with Clarity™ Western ECL substrate (1705060, Bio-rad) then imaged and analyzed using Image Lab software (Bio-Rad). Uncropped and non-modified images of representative Western blots can be found in Figure S4.

### 4.8. Matrigel Invasion Assay

Matrigel (Cat. 354230, Fisher Scientific, Waltham, MA) at 400 µg/mL concentration was placed in each transwell invasion chamber (8 µM pore, Cat. 3422, Corning, Waltham, MA, USA) and set overnight at 37 °C. A total of 8 × 10^4^ cells in complete growth medium were placed in the upper chamber and growth media supplemented with double the fetal bovine serum was placed in the lower chamber, then cells were allowed to invade for 18 h at 37 °C. For experiments using MK-2206, cells were incubated with MK-2206 overnight, then pre-incubated with the drug on ice for 30 min before plating in the upper chamber, which also contained the inhibitor. Non-invading cells were removed using a cotton swab, and transwell filters were fixed in methanol and stained with 4′, 6-Diamidino-2-Phenylindole, Dihydrochloride (DAPI) to visualize nuclei. Images of three random fields per chamber were obtained using the Nikon eclipse TE2000-U inverted microscope (Nikon Microscopy, Minoto city, Tokyo, Japan) and Fiji imageJ [62] was used to quantify the number of invaded cells.

### 4.9. Flow Cytometry

Cells were blocked in PBS/10% goat serum, then incubated with primary antibody against the integrin α3 subunit (MAB1952Z, EMD Millipore Corp, Burlington, MA, USA) or Normal mouse IgG (sc-2025, Santa Cruz Biotechnology, Dallas, TX, USA) at 5 µg/mL concentration, followed by secondary antibody, allophycocyanin, crosslinked, goat anti-mouse IgG ((REF: A865), Invitrogen) (1:200). Stained cells were fixed in 2% formaldehyde/PBS. Surface integrin α3β1 levels were measured using the FACSCalibur (Becton Dickinson, Franklin Lakes, NJ, USA) and analyzed using the FlowJo software (Becton Dickinson).

### 4.10. Bioinformatics

For survival analysis, cBioPortal was used to generate the Kaplan–Meier plot for patient tumors (Breast Invasive Carcinoma, TCGA, PanCancer Atlas) grouped as *BRN2* high and *BRN2* low (FPKM 0.1 cut-off, the Human Protein Atlas). To compare *BRN2* gene expression between breast cancer subtypes, we used the “explore selected study” option in cBioPortal for the Breast Invasive Carcinoma (TCGA, PanCancer Atlas) dataset and grouped patient tumors into hormone receptor-positive (Luminal A, Luminal B, HER2) and basal-like tumors. *BRN2* gene expression was then compared between groups. For *ITGA3* and *BRN2* mRNA correlation analysis, basal-like breast tumors from the Breast Invasive Carcinoma dataset (TCGA, PanCancer Atlas) were sorted by *ITGA3* expression and split into 2 groups as *ITGA3* high and *ITGA3* low. Using two-fold change and P-adj < 0.01 as the cut-off, we generated a list of *ITGA3*-correlated genes (results were obtained using DESeq2 in R). The bioinformatics results generated in this study utilized the TCGA Breast Invasive Carcinoma dataset generated by the TCGA Research Network (https://www.cancer.gov/tcga).

### 4.11. Statistics

All experiments were *n* ≥ 3. T-tests and Bonferroni corrections were applied as indicated in figure legends. For experiments using mice, each experimental data point was normalized to the average value of the control group.

## 5. Conclusions

Our work identifies a novel mechanism of integrin α3β1-mediated induction of the transcription factor, Brn-2, and links this regulation to invasive and metastatic properties of TNBC cells. Changes in the expression of transcription factors can affect global gene expression; it remains to be determined if the α3β1-dependent changes in Brn-2 expression can affect cancer cell properties beyond invasion and metastasis. Therefore, future research will investigate the breadth of α3β1-dependent Brn-2 effects in triple-negative breast cancer cells, towards further validating integrin α3β1 as a potential therapeutic target.

## Data Availability

Breast Invasive Carcinoma data set is available through cBioPortal (TCGA, PanCancer Atlas: https://www.cancer.gov/tcga).

## Supplementary Materials

The following are available online at https://www.mdpi.com/2072-6694/13/3/480/s1, Figure S1: Suppression of integrin α3β1 decreases Brn-2 protein in the TGL-4175 variant of MDA-MB-231 line, and in SUM159 cells, Figure S2: RNAi-targeting of integrin α3 or *BRN2* reduces SUM159 cell invasion, Figure S3. Suppression of integrin α3β1 or treatment with MK-2206 does not significantly alter total Akt protein levels in MDA-MB-231 cells. Figure S4: Uncropped Western blot images.
