# Supplementary material for: Integrin α3β1 Promotes Invasive and Metastatic Properties of Breast Cancer Cells through Induction of the Brn-2 Transcription Factor
Source: Cancers (Basel). 2021 Jan 27;13(3):480. doi: 10.3390/cancers13030480 (PMC7866210; doi:10.3390/cancers13030480)

cancers

MDPI

Supplemental materials: Integrin α3β1 promotes invasive and metastatic properties of breast cancer cells through induction of the Brn-2 transcription factor.

Rakshitha Pandulal Miskin[1], Janine S. A. Warren[2], Abibatou Ndoye[3], Lei Wu[3], John M. Lamar[2], and C. Michael DiPersio[2,3]*

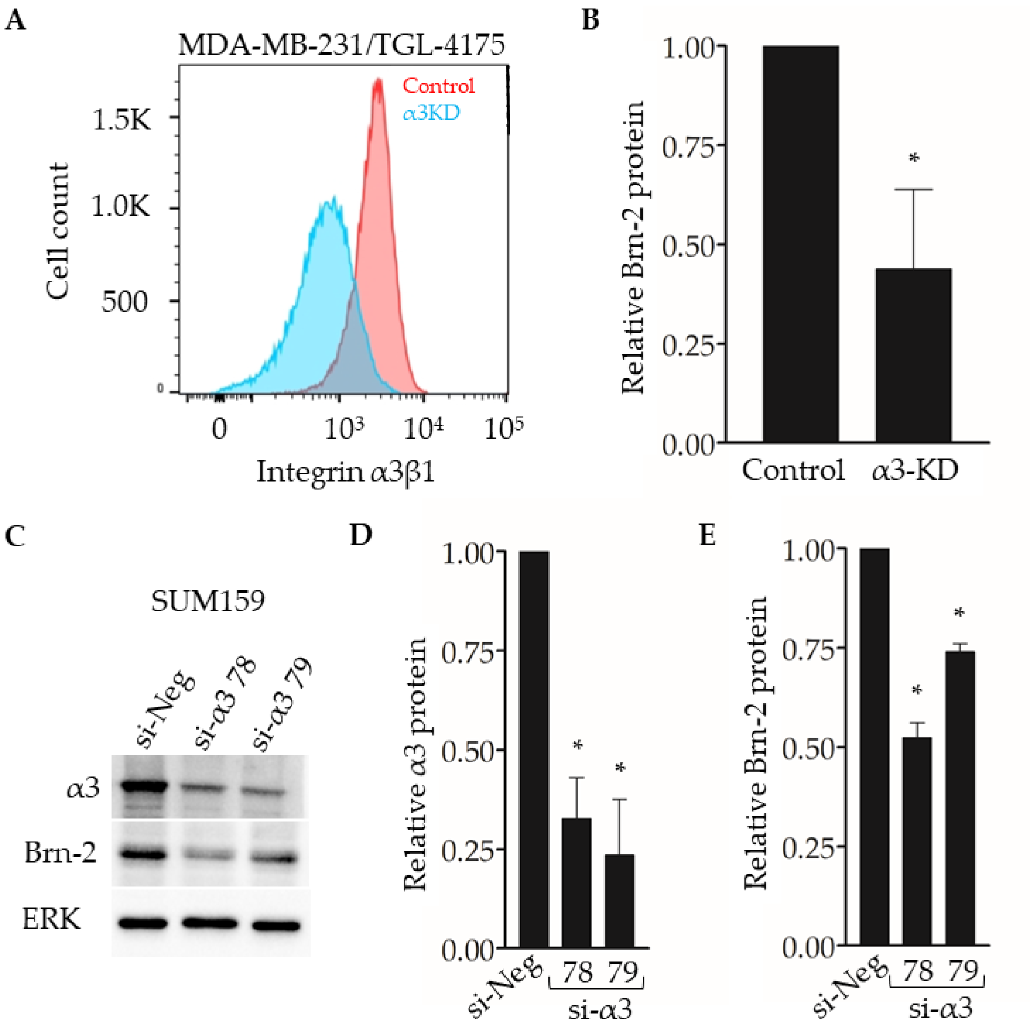

Figure S1. Suppression of integrin α3β1 decreases Brn-2 protein in the TGL-4175 variant of MDA-MB-231 line, and in SUM159 cells. (A) Flow cytometry using monoclonal antibody, P1B5, to compare cell surface levels of integrin α3 in TGL-4175 cells that stably express either non-targeting shRNA (Control) or α3-targeting shRNA (α3KD). (B) Western blot quantification of relative Brn-2 protein in α3KD TGL-4175 cells relative to control. (C) Representative western blots for integrin α3,

Brn-2 or ERK (loading control) in SUM159 cells transiently transfected with negative control siRNA (si-Neg) or with two distinct siRNAs that target integrin α3 (si-α3 78, si-α3 79). Data are quantified in (D) and (E), respectively, relative to control; n=3; mean +/- SEM; *p<0.05; two-tailed T-test (B) or two-tailed T-test with Bonferroni correction (D, E).

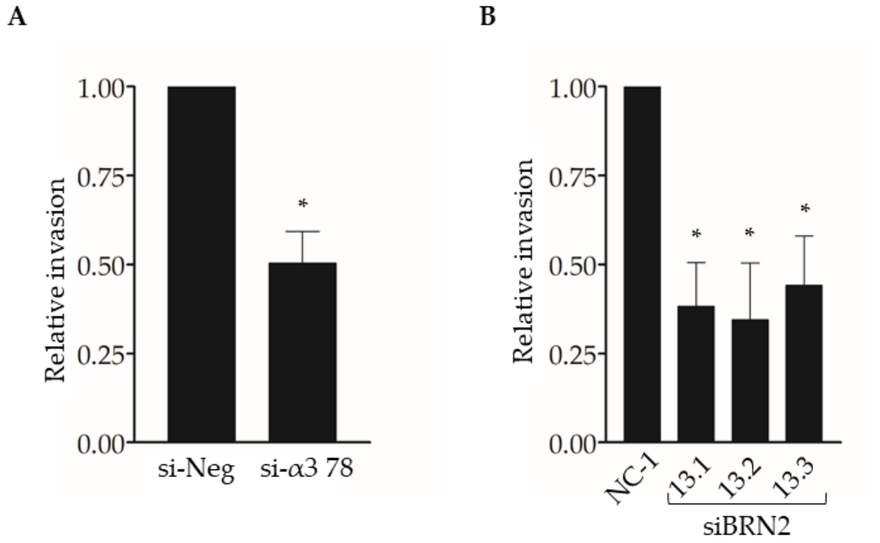

Figure S2. RNAi-targeting of integrin α3 or *BRN2* reduces SUM159 cell invasion. Matrigel transwell invasion assays were performed using SUM159 cells transfected with (A) non-targeting siRNA (si-Neg) or integrin α3-targeting siRNA (si-α3 78) and (B) non-targeting dicer-substrate siRNA (NC-1) or *BRN2*-targeting dicer-substrate siRNA (si*BRN2* 13.1, 13.2, or 13.3). Graphs show invasion of α3-knockdown cells (A) or Brn-2 knockdown cells (B) relative to control cells; n=3; mean +/- SEM; *p<0.05; two-tailed T-test (A) or two-tailed T-test with Bonferroni correction (B).

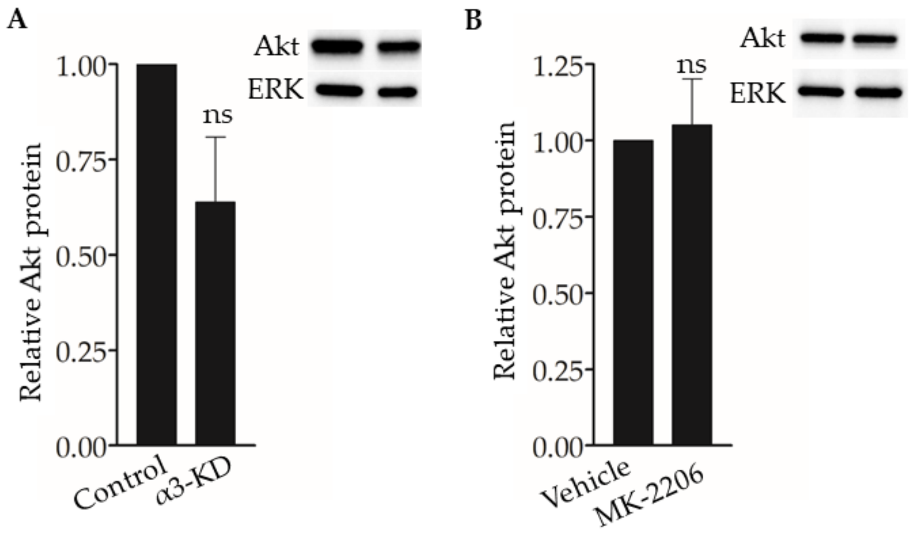

Figure S3. Suppression of integrin $\alpha 3\beta 1$ or treatment with MK-2206 does not significantly alter total Akt protein levels in MDA-MB-231 cells. (A, B) Representative western blots and corresponding graphs show Akt levels in (A) α3KD cells relative to control cells, and (B) MDA-MB-231 cells treated with MK-2206 relative to vehicle-treated cells; n=3; mean +/- SEM; *p<0.05; two-tailed T-test.

Figure S4: Uncropped Western blotting figures

Integrin $\alpha3\beta1$ promotes invasive and metastatic properties of breast cancer cells through induction of the Brn-2 transcription factor

Rakshitha Pandulal Miskin[1], Janine S. A. Warren[2], Abibatou Ndoye[3], Lei Wu[3], John M. Lamar[2], and C. Michael DiPersio[2,3]*

Figure 3A

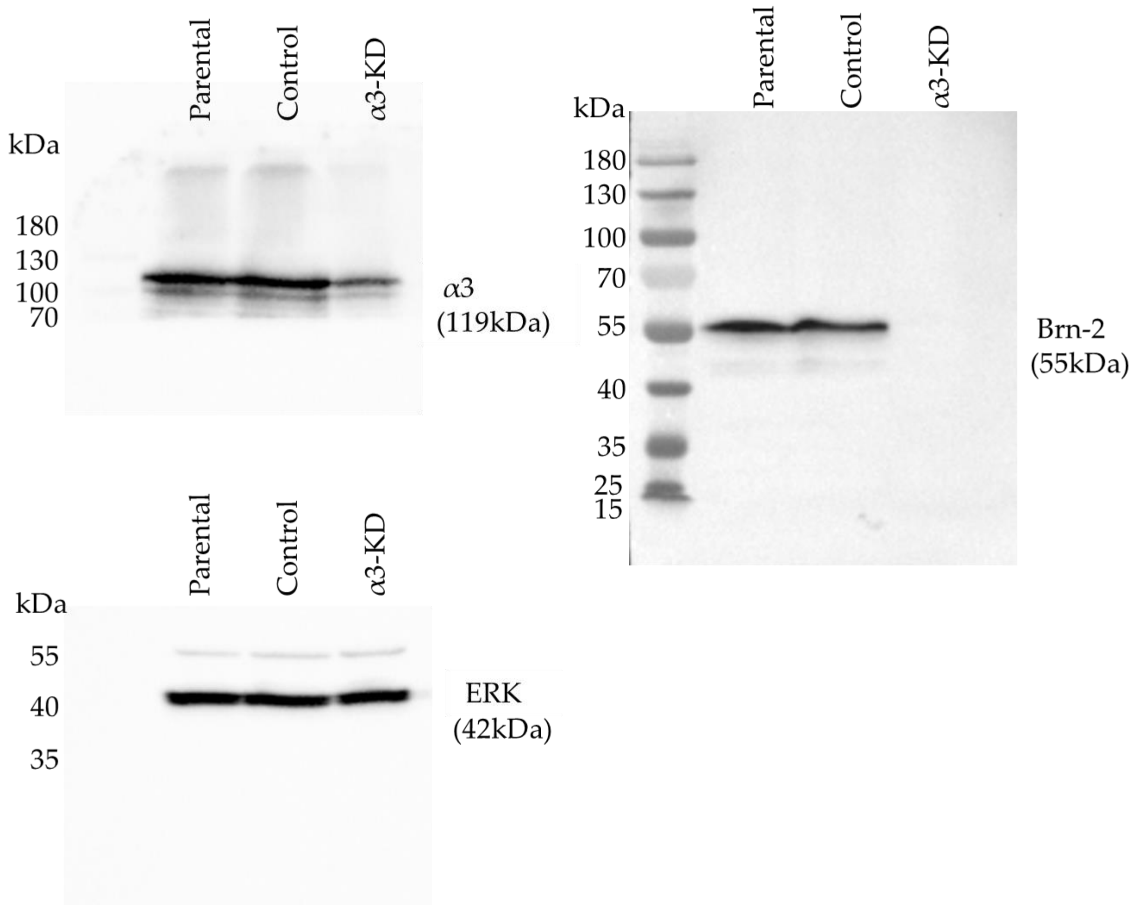

Figure 3C

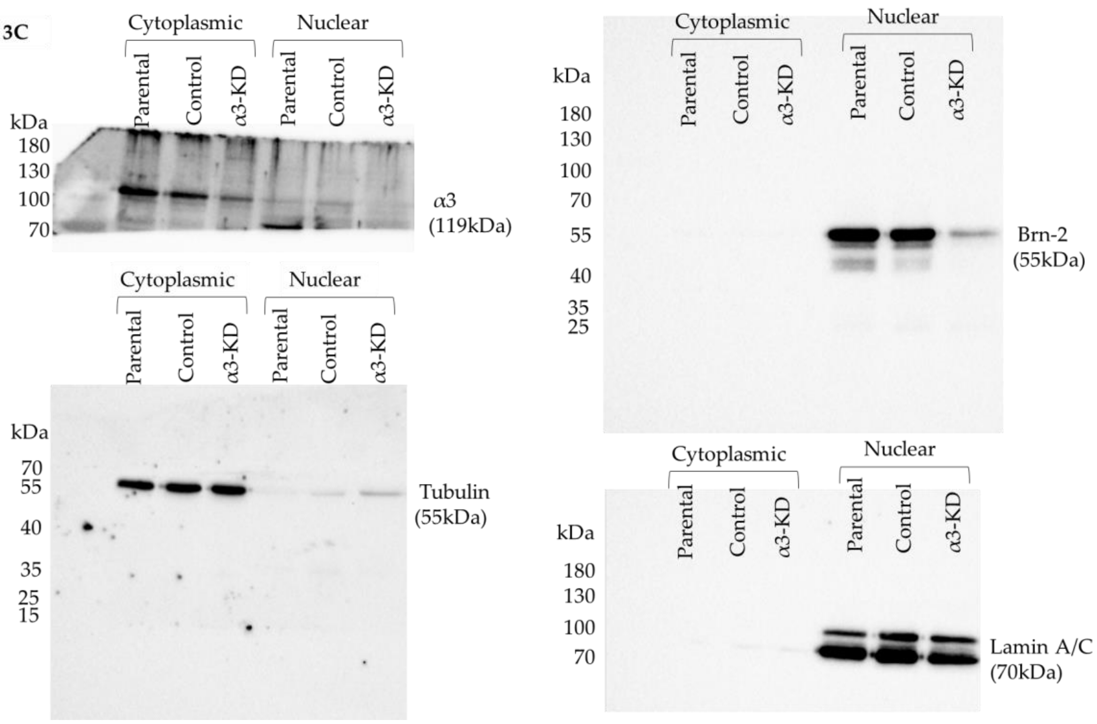

Figure S1C

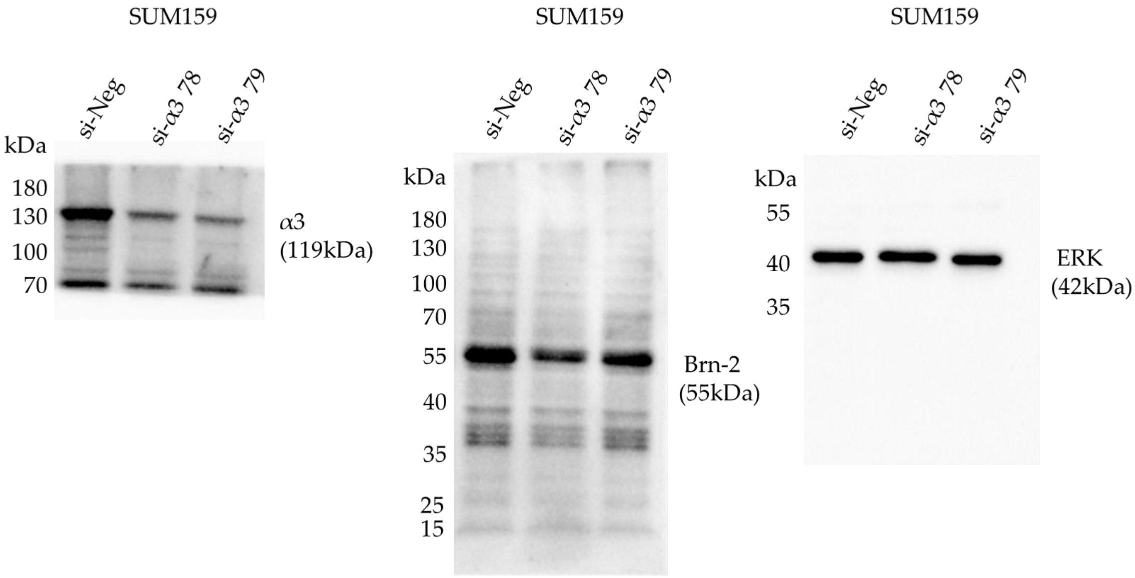

Figure 5A

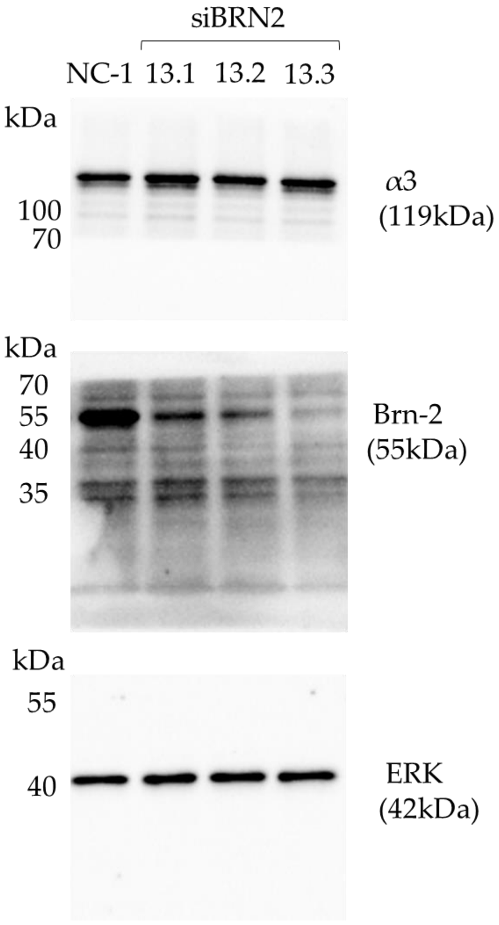

Figure 7A

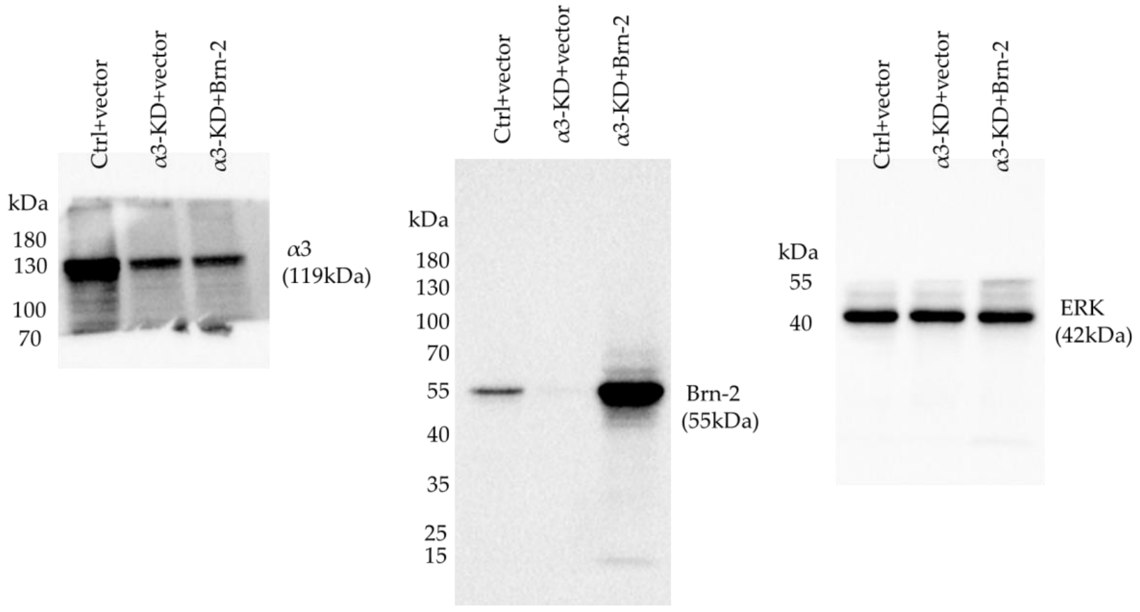

Figure 8A

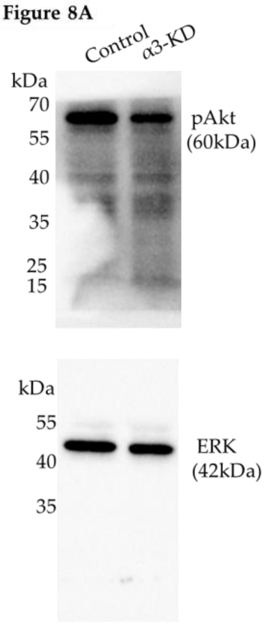

Figure 8B

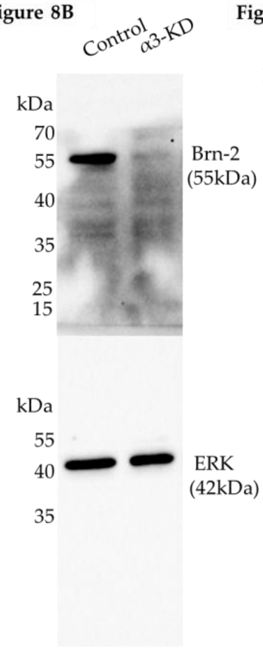

Figure 8C

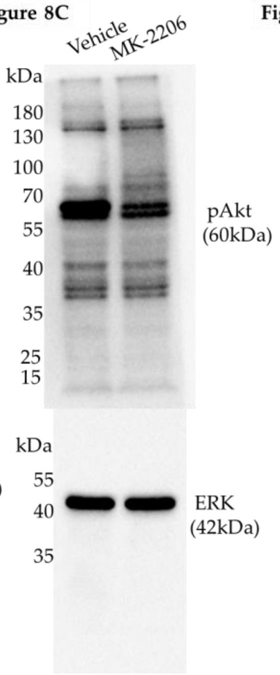

Figure 8D

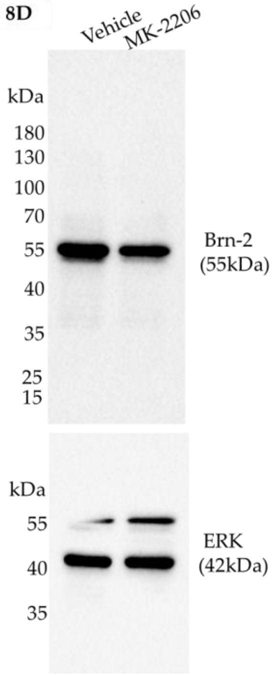

Figure S3A

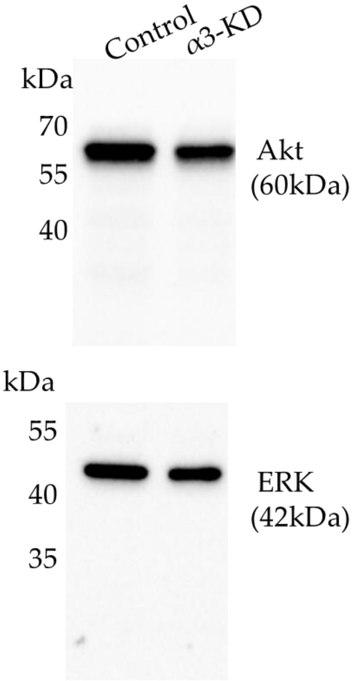

Figure S3B

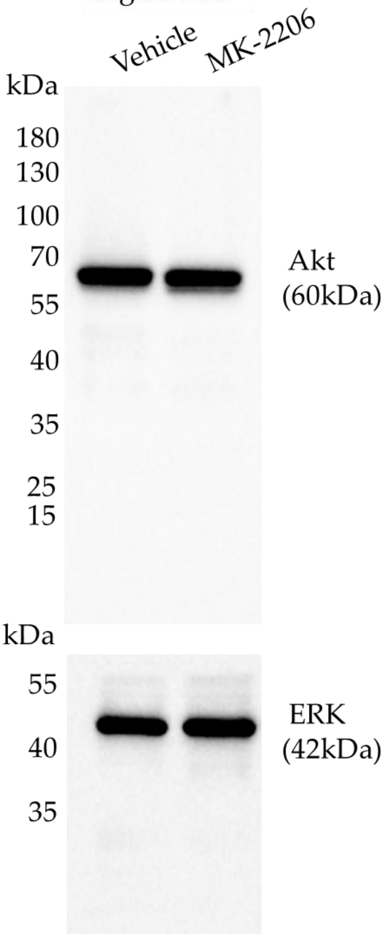

Supplement: Supplementary file 1 [file cancers-13-00480-s001.pdf]
